# Successful cases of percutaneous left ventricular assist device “Impella” to fulminant myocarditis

**DOI:** 10.1186/s13019-022-01821-x

**Published:** 2022-04-12

**Authors:** Takayuki Hori, Mitsuru Iida, Masateru Uchiyama, Tomoki Shimokawa

**Affiliations:** 1 Department of Cardiovascular Surgery, Teikyo University Hospital, 2-11-1 Kaga, Itabashi-ku, Tokyo, 173-8605 Japan; 2 Department of Cardiovascular Surgery, The Cardiovascular Institute, Tokyo, Japan

**Keywords:** Fulminant myocarditis, Percutaneous left ventricular assist device, Impella

## Abstract

**Background:**

Fulminant myocarditis (FM) is a form of severe inflammatory carditis with rapidly developing acute heart failure.

**Case presentation:**

We report three cases of successful intensive treatment by Impella of FM without any complications. In all cases, impairment of microcirculation as measured by blood lactate level and the hemodynamic value as indicated by cardiac index were improved within 24–48 h and 7 days after Impella implantation, respectively. Interestingly, our data also suggested that treatment by Impella CP or 5.0 may lead to faster recovery of microcirculation and cardiac function than treatment by Impella 2.5.

**Conclusion:**

Our findings demonstrate that the appropriate selection of Impella devices guided by body surface area measurements may help to improve clinical outcomes of severe heart failure including FM.

## Background

Percutaneous left ventricular assist device “Impella” (Abiomed, Danvers, MA) is a microaxial pump system [1], which can be inserted without thoracotomy and was first covered by health insurance in September 2017 in Japan. Impella has since been considered a highly regarded treatment strategy due to its utility of circulatory support [2, 3]. We have previously demonstrated findings from the first case using Impella in Japan to a patient who suffered from ventricular septal perforation after acute myocardial infarction [4]. In our case series, we report three cases of Impella to patients with fulminant myocarditis (FM), severe inflammatory carditis with rapidly developing acute heart failure, cardiogenic shock, and fatal arrhythmia.

## Case presentation

### Case 1

**Fig. 1:**
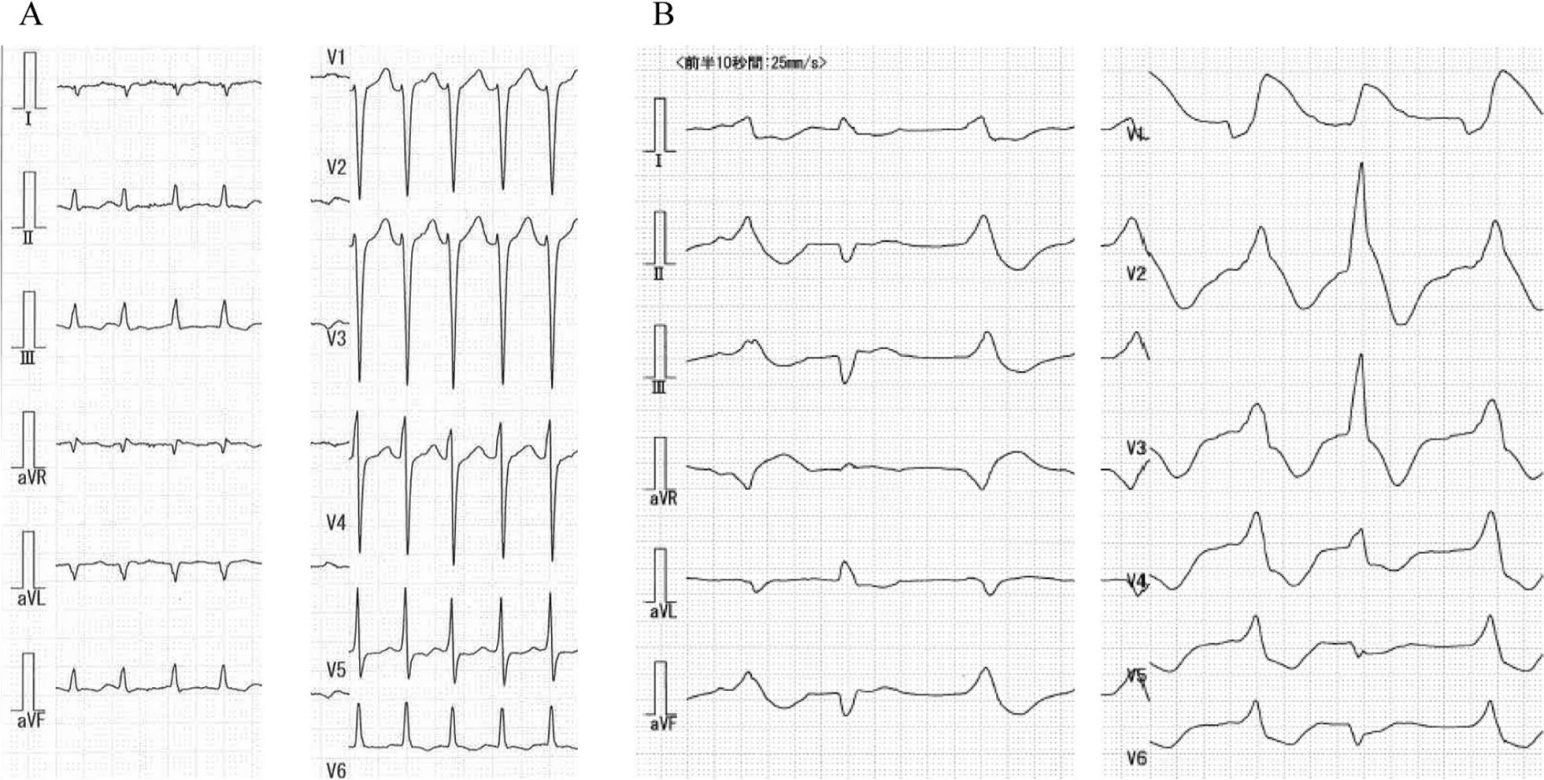
Electrocardiogram before Impella implantation. **A** Severe tachycardia in Case 1. **B** Ventricular tachycardia in Case 2

**Fig. 2:**
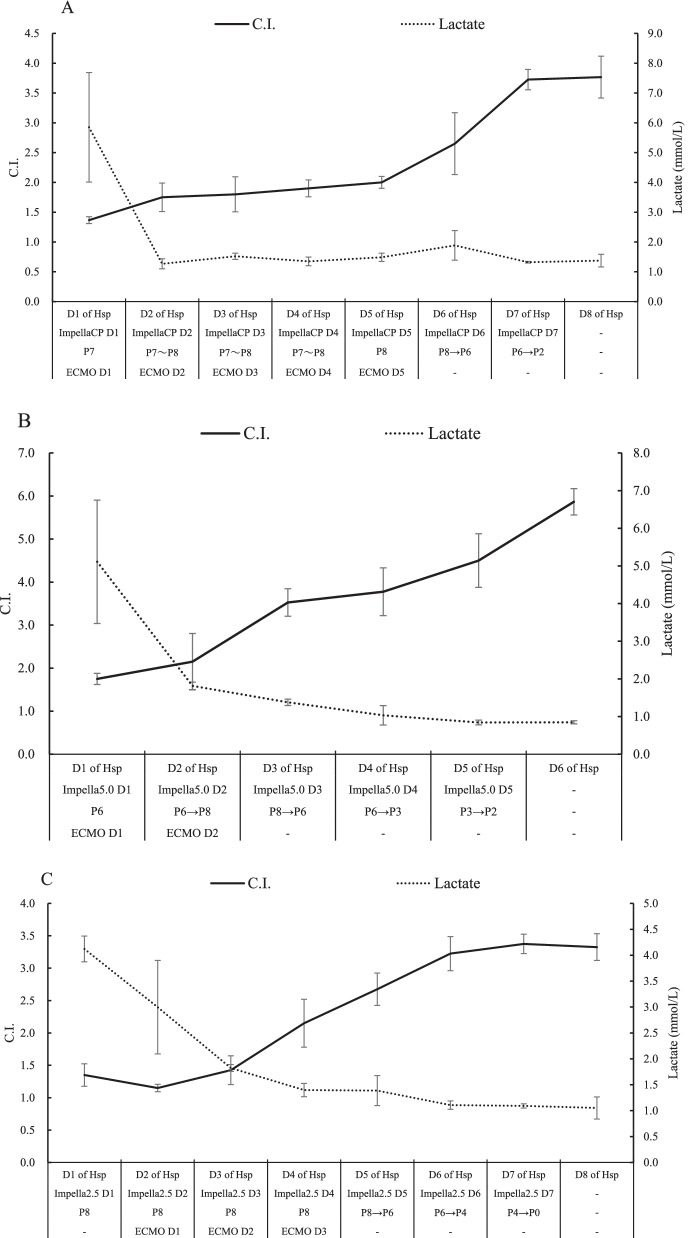
Clinical findings after Impella implantation and management of ventricular assist device. Transition of the level of lactate and cardiac index in the clinical course of Case 1 (**A**), Case 2 (**B**), and Case 3 (**C**). C.I., cardiac index

A 22-year-old man presented fever that had begun 5 days before and had taken antibiotics (Table 1). The patient had no past medical history. On admission, electrocardiogram showed severe tachycardia (Fig. 1A), and a blood test showed high level of lactate (Table 2). Transthoracic echocardiography (TTE) revealed impaired left ventricular function (left ventricular ejection fraction (LVEF): 15%) (Table 3). In addition, Coxsackievirus A was detected by blood culture. The patient was transferred to our hospital for detailed analysis and treatment of fulminant myocarditis. Due to unstable vital signs under high doses of inotropic agents, Impella CP (assist flow: 2.439 L/min/m^2^) was implanted to the right femoral artery (Table 1). All the hemodynamic values improved within the first 48 h of Impella support. After Impella implantation, extracorporeal membrane oxygenation (ECMO) was inserted because of impaired right ventricular function. Immediately after treatment of Impella and ECMO, the level of lactate significantly improved. Inotropic agents were reduced gradually, and ECMO was removed on the 5th day after insertion, and Impella was removed after another 5 days (Fig. 2A). LVEF improved to 52% after Impella removal (Table 3). The recovery of symptoms and postoperative blood tests progressed satisfactorily. The patient was discharged on day 19 without serious complications.

**Table 1:** Patients characteristics

	Case 1	Case 2	Case 3
Age (years)	22	16	68
Gender	Male	Male	Male
Height (cm)/weight (kg)	160/45	176/65	156/48
Body surface area (m^2^)	1.44	1.80	1.45
Type of Impella	CP	5	2.5
Approach	Femoral	Axillary	Femoral
Cause of fulminant carditis	Coxsackievirus A	Coxsackievirus A4	Unknown

**Table 2:** Blood test on admission

	Case 1	Case 2	Case 3
WBC (µL)	20,000	10,900	20,000
AST (U/L)	11,496	2627	1444
CK (IU/L)	1568	5465	3721
CK-MB (ng/mL)	127	110	140
LDH (U/L)	10,781	4130	1545
Lactate (mmol/L)	9.5	9.3	4.0

**Table 3:** Transition of transthoracic echocardiography data

	Case 1	Case 2	Case 3
LVEF (%)			
Pre-Impella	15	14	27
Post-Impella	52	59	46
LVDd (mm)			
Pre-Impella	32	48	54
Post-Impella	47	52	44
LVDs (mm)			
Pre-Impella	28	44	47
Post-Impella	32	33	34

LVEF, left ventricular ejection fraction; LVDd, left ventricular end-diastolic diameter; LVDs, left ventricular end-systolic diameter

### Case 2

A 16-year-old man with no past medical history presented sudden fever, chest pain, and loss of consciousness, resulting in cardiopulmonary arrest (Table 1). The patient was transferred to another hospital in a state of cardiopulmonary arrest. ECMO and intra-aortic balloon pump were inserted and while return of spontaneous circulation was confirmed, ventricular tachycardia (VT) was not controlled (Fig. 1B). The patient was transferred to our hospital for a more detailed analysis. A blood test on admission showed high level of creatinine kinase and lactate (Table 2). TTE revealed impaired left ventricular function (LVEF: 14%) (Table 3). Despite high doses of inotropic agents, VT storm was difficult to control. In addition, Coxsackievirus A4 was detected by blood culture, and the patient was diagnosed with fulminant myocarditis. Due to unstable vital signs and drug-resistance arrhythmia, Impella 5.0 (assist flow: 2.793 L/min/m^2^) was implanted via prosthetic graft to the right axillary artery after removal of intra-aortic balloon pumping and insert of ECMO (Tables 1, 2, 3). All the hemodynamic values and lactate levels improved within the first 48 h of Impella support. Impella was removed 5 days after implantation (Fig. 2B). LVEF improved to 59% after Impella removal (Table 3). The patient started cardiac rehabilitation and was discharged on day 23 without serious complications.

### Case 3

A 68-year-old man with no medical history presented cold-like symptoms and mild breathlessness that had begun a couple of days before; however, the symptoms did not improve. Initially suspected of asthma and hepatic dysfunction, the patient was admitted to our hospital (Table 2). TTE on admission revealed significantly impaired ventricular function (LVEF: 27%) (Table 3). Unfortunately, any causative microorganisms were not detected by blood culture, and we diagnosed fulminant myocarditis based on the clinical course and examination findings. Because the patient was in cardiogenic shock on the first day of admission, Impella 2.5 (assist flow: 1.722 L/min/m^2^) was implanted to the right femoral artery (Table 1). ECMO was used because the hemodynamics supported by Impella and recovery of peripheral circulation were insufficient (Fig. 2C). As respiratory condition gradually improved, ECMO and Impella were removed on day 3 and 7, respectively. LVEF improved to 46% after Impella removal (Table 3). The patient started cardiac rehabilitation and was discharged on day 59 without serious complications.

## Discussion

FM is a well-known severe inflammatory myocardial disease with rapidly developing heart failure, cardiac shock, and life-threatening arrhythmia. Generally, FM requires intensive treatments including mechanical circulatory support, and heart transplantation in some cases [5]. We describe three cases of successful intensive treatment by Impella of fulminant myocarditis without any complications. At the time of cardiogenic shock on admission, right ventricular function in all cases were relatively normal; however, indication for ECMO was required because of the presentation of right ventricular failure or deterioration of respiratory condition in the clinical course of all cases. Although there is a slight time gap, impairment of microcirculation with blood lactate level as an index was improved within 24–48 h. Additionally, the hemodynamic value with cardiac index (CI) as an index was improved within 7 days after Impella implantation. Although a direct comparison cannot be made, our data suggested that a case treated by Impella 2.5 may have slower recovery of microcirculation than other cases treated by Impella CP or 5.0. Additionally, the case treated by Impella 2.5 had the longest hospital stay (59 days). As one of the possibilities, the poor outcomes by Impella 2.5 may be a result of body surface area (BSA) mismatch and consequently insufficient unload. To maintain 2.2 L/min/m2 of CI needed to improve heart failure, a proper Impella device based on BSA should be selected. For example, in cases with < 1.14 of BSA, 1.15–1.6, and > 1.6, Impella 2.5, CP, and 5.0 need to be used for securing 2.2 L/min/m^2^ of CI, respectively. Our data demonstrated that Case 1 with Impella CP (BSA 1.44) and Case 2 with Impella 5.0 (BSA 1.80) had satisfactory improvement of cardiac function. However, Case 3 (BSA 1.45) treated by Impella 2.5 had slower recovery of cardiac function (Fig. 2C). Taking into account the degree of unloading in this case, Impella 5.0 may have been the more advisable choice. Nevertheless, the fact that Impella 5.0 takes longer for implantation is also worth consideration in these cases. Taken together, this case series may be a good implication when we wonder which device to indicate in patients with a small physique.

## Conclusion

Our findings regarding three cases of Impella treatment to patients with FM indicate that the proper selection of Impella devices according to BSA measurements may help to improve clinical outcomes of severe heart failure including FM.

## Data Availability

The datasets used are available from the corresponding author on reasonable request.

## Abbreviations

ECMO: Extracorporeal membrane oxygenation
LVEF: Left ventricular ejection fraction
FM: Fulminant myocarditis
TTE: Transthoracic echocardiography
VT: Ventricular tachycardia
